# C-952-03. Burn Simulation: A Burn Center Quality Improvement Tool

**DOI:** 10.1093/jbcr/irag033.119

**Published:** 2026-04-06

**Authors:** Michaela M Craig, Sarah Wright, Christopher K Craig, Jeffrey Williams, James H Holmes, IV

**Affiliations:** Atrium Health Wake Forest Baptist Burn Center, Winston-Salem, NC; High Point University, High Point, NC; Atrium Health Wake Forest Baptist Hospital / High Point University, High Point, NC; High Point University Department of Physician Assistant Studies, High Point, NC; Wake Forest University School of Medicine, Winston-Salem, NC

## Abstract

**Introduction:**

Burns are a common injury, with over 500 000 people seeking treatment and causing approximately 4000 deaths each year in the United States. Caring for burns is complex, and effective management requires extensive knowledge, skill, and efficiency to minimize morbidity and mortality in burn patients. Artificial simulation, through immersive training, provides a realistic representation of complex situations and has been successfully implemented across various medical fields, improving healthcare providers' techniques without the risk of patient harm.

**Methods:**

High-fidelity simulations were conducted using either standardized patients or manikins, each incorporating a 5-minute pre-brief, a 15-minute simulation, and a 15-minute debrief. This qualitative study was conducted at an ABA-verified burn center in collaboration with their in-house Center for Experimental and Applied Learning (CEAL). Following each scenario, both participants and observers completed a post-simulation survey consisting of seven Likert-style questions (5-point scale).

**Results:**

A total of 26 surveys were collected from 14 participants and 12 observers across three simulation events. Average scores for each of the seven questions ranged from 4.6 to 4.9 out of 5. The mean total score for all surveys was 4.7 out of 5. A Mann–Whitney U test revealed no statistically significant difference in total or individual question scores between observers and participants (U = 48, z = -1.96, *p*=.07).

**Conclusions:**

Survey responses demonstrated strong positive feedback, and one simulation scenario highlighted a procedural gap that led to a subsequent quality improvement initiative. Nursing staff were highly engaged and receptive to simulation-based learning. As a single-center pilot study, additional research is recommended to explore the broader applicability of these findings and to evaluate long-term outcomes associated with simulation training in burn care.

**Applicability of Research to Practice:**

High-fidelity burn simulations provide a safe, effective way to identify gaps in practice, strengthen provider skills, and improve team communication without patient risk. Incorporating simulation into routine burn center education can standardize care and enhance outcomes for burn patients.

**Funding for the study:**

N/A.

